# Understanding vaccine recommendation behaviours among healthcare workers in Senegal: A cross‐sectional analysis

**DOI:** 10.1111/tmi.70002

**Published:** 2025-06-29

**Authors:** Sébastien Cortaredona, Pierre Verger, Jean Constance, Aldiouma Diallo, El‐Hadj Ba, Gwenaelle Maradan, Cheikh Sokhna, Patrick Peretti‐Watel

**Affiliations:** ^1^ Aix‐Marseille Univ, IRD, SSA, MINES Marseille France; ^2^ Aix Marseille Univ, SSA, RITMES Marseille France; ^3^ IHU‐Méditerranée Infection Marseille France; ^4^ Unité des Virus Émergents (UVE: Aix‐Marseille Univ, Università di Corsica, IRD 190, Inserm 1207, IRBA) Marseille France; ^5^ Observatoire régional de la santé PACA (ORS Paca), Aix‐Marseille Université Marseille France; ^6^ Comité national d'éthique pour la recherche en santé (CNERS) Dakar Senegal; ^7^ Conseil consultatif sur les vaccins au Sénégal (CCVS) Dakar Senegal; ^8^ IRD, MINES, Campus International IRD‐UCAD Dakar Senegal

**Keywords:** healthcare professionals, Senegal, sub‐Saharan Africa, vaccine confidence, vaccine hesitancy, vaccines

## Abstract

**Background:**

Despite the pivotal role of healthcare workers in vaccination programs, there is limited understanding of the factors influencing their vaccine recommendations, particularly in low‐resource and rural settings. This study examines the determinants of vaccine recommendation practices among healthcare workers in Senegal.

**Methods:**

A cross‐sectional survey was conducted in 2024 among 302 healthcare workers in Senegal. A vaccine recommendation score was constructed to assess how frequently healthcare workers recommended vaccines. A typology of psychosocial determinants of healthcare workers' vaccination behaviour was developed using the short version of the Health Professionals Vaccine Confidence and Behaviours questionnaire. Multivariable log‐binomial regression was used to identify factors associated with systematic vaccine recommendations.

**Results:**

Vaccine recommendation practices among healthcare workers were high, with 60.6% achieving the highest score. The Professionals Vaccine Confidence and Behaviours typology classified healthcare workers into three clusters: ‘Highly confident’ (57.3%), ‘Moderately hesitant’ (14.2%), and ‘Specific hesitant’ (28.5%). Healthcare workers with more than 3 years of experience and those in urban areas were significantly more likely to systematically recommend vaccines. Conversely, healthcare workers displaying higher complacency, lower openness to patients, reduced commitment to vaccination, and limited self‐efficacy were less consistent in their recommendations.

**Conclusion:**

While healthcare workers in Senegal demonstrate high vaccine confidence and vaccine recommendation practices, disparities between urban and rural settings highlight the need for targeted interventions. Efforts should focus on enhancing training, resources, and support for healthcare workers in rural areas to address barriers and strengthen vaccine promotion. Future research should explore contextual factors shaping healthcare workers' vaccination attitudes and practices to inform tailored strategies for equitable vaccination uptake.

## INTRODUCTION

**FIGURE 1:**
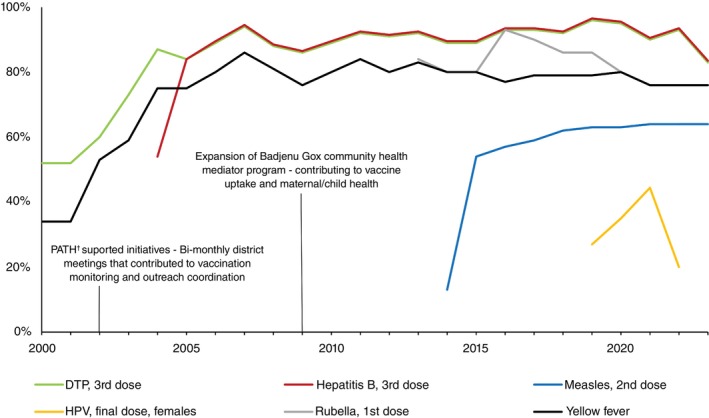
Senegal Vaccination coverage trendline. Source: Estimates of National Immunisation Coverage (WUENIC) for Senegal. https://immunizationdata.who.int/dashboard/regions/african‐region/SEN . ^†^PATH (Program for Appropriate Technology in Health) is an international nonprofit organisation that supports health innovations to improve equity.

Vaccination is one of the most powerful and cost‐effective public health interventions, saving millions of lives each year [1]. Despite its effectiveness in reducing infectious disease‐related morbidity and mortality, under‐vaccination and non‐vaccination remain major challenges, particularly in Africa [2]. Around 1 in 5 children in Africa lack basic vaccines, resulting in over 30 million children under five suffering from vaccine‐preventable diseases (VPD) annually, with more than half a million dying—accounting for 58% of global VPD‐related deaths [3]. In Senegal, after a decline in vaccination coverage for measles, polio, yellow fever, and maternal and neonatal tetanus in 2020 during the COVID‐19 pandemic, vaccination rates have shown significant variability across vaccines and regions, with some falling short of national and international coverage targets [4]. While coverage for the third dose of the diphtheria‐tetanus‐pertussis vaccine was relatively high in 2023 (83%), measles coverage for the second dose was only 64%, well below the 95% needed to achieve herd immunity (Figure 1) [5]. The situation is even more concerning for the human papillomavirus (HPV) vaccine, with only 20% of eligible girls completing the second dose—far short of the WHO target of 90% coverage by 2030 [5]. Given the increasing number of vulnerable older adults with comorbidities in Senegal, as in many low‐ and middle‐income countries, there is a growing need to extend immunisation programmes beyond childhood, recognising that vaccination remains a critical public health intervention across all age groups [6, 7].

As in many countries in sub‐Saharan Africa (SSA), healthcare workers (HCWs) play a critical role in promoting vaccination in Senegal [8, 9]. As trusted community figures, they serve as the first point of contact for parents and children, positioning them to significantly influence vaccine acceptance [10]. They collaborate closely with schools, community leaders, and public health officials to ensure the smooth delivery of vaccines and encourage completion of full vaccination schedules [10, 11, 12]. In Senegal, initiatives such as the Badjenu Gox program, which mobilises respected women from the community to foster trust between healthcare providers and families, and the Program for Appropriate Technology in Health (PATH), which has supported bi‐monthly district‐level review meetings, have contributed to strengthening vaccination efforts and improving immunisation coverage in the country (Figure 1). A distinctive feature of Senegal's Expanded Program on Immunisation (EPI) is the active involvement of civil society, particularly through community mediators. Another key feature of Senegal's EPI is its dual approach: health centers administer vaccines to children in urban areas and villages within about 15 kilometres of a health center, while mobile teams [13] ensure vaccine delivery to children in more remote villages where much of the population still lives [14, 15].

Trained and motivated HCWs are crucial to the success of Senegal's vaccination program, as they play a key role in improving vaccine acceptance, dispelling misconceptions and addressing vaccine hesitancy among their patients [2, 16]. However, this vaccine hesitancy, defined by the WHO Behavioural and Social Drivers of Vaccinations working group as “*A motivational state of being conflicted about, or opposed to, getting vaccinated; includes intentions and willingness*.” [17], also affects HCWs. Vaccine hesitancy among HCWs can not only influence their personal vaccine uptake but also impact their vaccine recommendations (or lack thereof) to their patients and remains a complex issue [18, 19, 20]. Research has demonstrated that HCWs who possess accurate knowledge of vaccines, understand the risks of vaccine‐preventable diseases, and trust in vaccine effectiveness are more likely to recommend them to their patients [18, 19, 21, 22]. On the other hand, misinformation and doubts about vaccine safety can negatively impact their recommendations. Additionally, HCWs in rural areas often face additional barriers, including a lack of training and exposure to widespread myths and misconceptions in their communities [2].

This study aims to investigate the determinants of vaccine recommendation behaviours among HCWs in Senegal, a critical yet understudied population in the context of vaccination promotion, particularly in rural settings. Using a cross‐sectional design, we surveyed HCWs from both urban and rural areas to assess their vaccine recommendation practices and explore psychosocial factors influencing these behaviours, employing a validated instrument—the Health Professionals Vaccine Confidence and Behaviours (Pro‐VC‐Be) questionnaire [23]. Our objective was to identify key individual and contextual determinants associated with systematic vaccine recommendations, with the goal of informing targeted interventions to strengthen vaccination promotion strategies and, ultimately, improve vaccination coverage across diverse healthcare settings in Senegal.

## METHODS

### Population and setting

**FIGURE 2:**
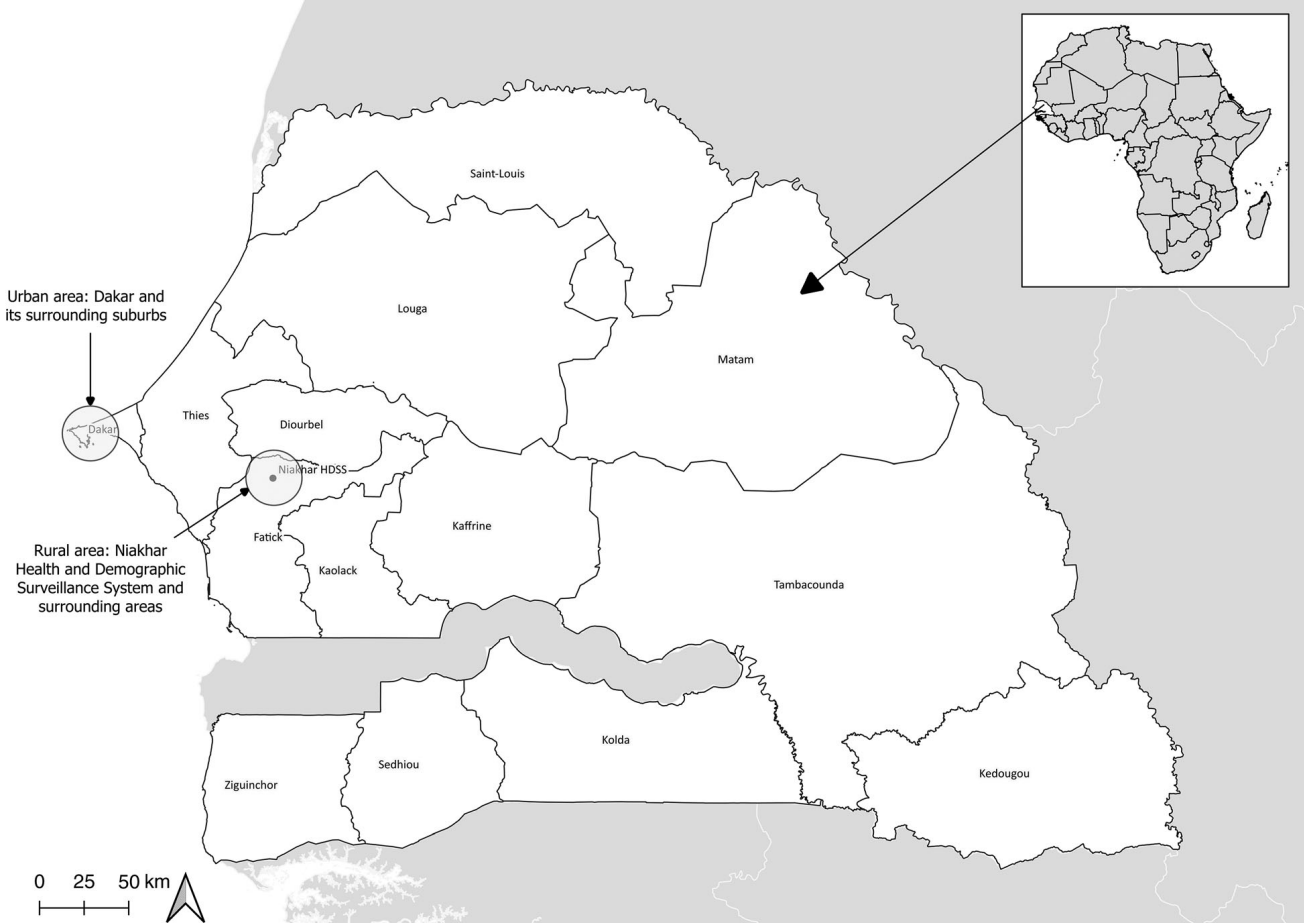
Map of the study areas.

We conducted a cross‐sectional survey among HCWs in two distinct regions of Senegal: one urban (Dakar and its surrounding suburbs) and one rural (Figure 2). The rural area included the region covered by the Niakhar Health and Demographic Surveillance System (HDSS), located in the Niakhar district of the Fatick region, approximately 135 km southeast of Dakar [24]. The Niakhar HDSS covers an area of 203 km^2^ and had a population of 50,355 as per the January 2018 census [25]. The primary economic activity in this region is agriculture and the majority of the population belongs to the Serere ethnic group. Our initial goal was to survey 200 HCWs in the rural area and 100 HCWs in the urban area. Due to challenges in recruiting 200 HCWs in the rural area, the survey zone was expanded to include areas surrounding the Niakhar HDSS, while still remaining within the Fatick region (Figure 1). In the rural area, all health posts were included due to their limited number. In the urban area, health posts were randomly selected from a list provided by the Dakar regional health office (*Direction régionale de la santé de Dakar*) until the target of at least 100 HCWs was reached. Prior to the field survey, the heads of the health posts in the survey areas were contacted and informed about the objectives of the study. A few days later, professional and trained interviewers visited the health posts to conduct face‐to‐face interviews with all HCWs involved in vaccination who were present at the health post. This included: nurses, head/major nurses, nursing assistants, midwives, midwife assistants, community HCWs and Badjenu Gox—respected women who serve as mediators between healthcare providers and the community [26]. These interviews were conducted using tablet PCs in French or Serer, depending on the participant's language preference. Data collection began in December 2023 and concluded in May 2024. Overall, 99.0% (302/305) of eligible HCWs were interviewed.

### Psychosocial determinants of healthcare workers' vaccination behaviour

To measure vaccine confidence and other psychosocial determinants of HCWs vaccination behaviour, we used the short version of the Pro‐VC‐Be instrument [23] that we adapted to the context of Senegal. The Pro‐VC‐Be is grounded in three prominent theoretical frameworks [20]: the Theoretical Domains Framework—a consensus approach for the development of a theoretical framework of constructs that may be relevant in vaccine‐related intervention studies of HCWs [27], the Health Belief Model—which postulates that the adoption of preventive behaviours depends on their perceived benefits and risks, considered in light of the disease's perceived severity [28], and the 5C model for vaccine hesitancy [29]. The adapted version of the Pro‐VC‐Be measures 10 key dimensions of psychosocial determinants related to HCWs vaccination behaviour (see Data S1 for the English version). To adapt the short version of the Pro‐VC‐Be questionnaire, which was developed and validated in French‐speaking countries, we consulted Senegalese doctors who are experts in vaccination to review each item. Following these discussions, several modifications were made to better fit the Senegalese context. Prior to the survey, we conducted a cognitive validation with a small sample of five HCWs from different professional backgrounds to ensure that the way the questions were understood corresponded to their intended meaning in the Pro‐VC‐Be questionnaire. This validation was conducted in both French and Serer to ensure clarity across different languages.

### Outcome

The questionnaire asked HCWs how frequently they use to recommend the following vaccines using a 5‐point Likert scale (Never to Always, with a ‘Don't know’ option): tetanus, measles/rubella/yellow fever, hepatitis B, HPV vaccine (for mothers of girls aged 9 to 14), and COVID‐19 vaccines (for adults under 60 and adults over 60). Additionally, a ‘Do not receive/visit this type of patient’ option was provided for each item to accommodate different care settings. With a Cronbach's alpha of 0.78, indicating good internal consistency, a vaccine recommendation score was calculated by conducting a multiple correspondence analysis (MCA) [30]. Prior to MCA, all items were binarized into “Always” and “Not always” categories. Responses such as ‘Don't know’ and “Do not receive/visit this type of patient” were imputed using the MCA model.

### Covariates

In addition to socio‐demographic variables (sex and age), we collected the following information for each participant: area of practice (urban [Dakar]/rural [Fatick]), position (community HCWs/Badjenu Gox, nurse/nursing assistant, head nurse/major nurse, midwife/midwife assistant, other), tenure / experience in the current position (<3 years, 3–9 years, and >9 years), professional status (civil servant, permanent contract, fixed‐term contract, volunteer), number of patients per week (<50, 51–100, >100), estimated workload (low, average, high, and too high), and attendance at vaccination seminars in the past 3 years (no, once, more than once).

### Statistical analysis

An MCA combined with agglomerative hierarchical cluster analysis (HCA) was performed on items from the adapted short version of the Pro‐VC‐Be questionnaire to assign HCWs to data‐driven clusters [31]. We conducted bivariate analyses to identify factors, including the Pro‐VC‐Be typology, associated with the vaccine recommendation score. Since the majority of HCWs achieved the maximum score, we binarized it and used Fisher's exact tests for associations. We then applied a log‐binomial multivariable regression model with an automatic stepwise selection procedure (*p* < 0.05), incorporating factors with *p* < 0.10 from the bivariate analysis. All analyses were conducted using two‐sided *p*‐values, with statistical significance at *p* ≤ 0.05. Statistical analyses were conducted with SAS 9.4 (SAS Institute, Cary, NC).

### Ethical considerations

Each participant received an information leaflet, informing them that the survey was anonymous and the data collected confidential, and that she could withdraw from the study at any time, both during and after completion of the questionnaire. Each participant signed a written consent form. The survey's protocol was approved by the Senegalese National Ethical Committee for Research in Health (N° 00000216/MSAS/CNERS/SP, renewed on September 18, 2023, N°000289/MSAS/CNERS/SP).

## RESULTS

### Participants' characteristics

The study surveyed 302 HCWs, predominantly women (251/302, 83.1%), with an average age of 38.6 years (±9.1) (Table 1). Most respondents (173/302, 57.3%) were from rural areas. The largest group comprised nurses or nursing assistants (115/302, 38.1%), followed by community health workers, including Badjenu Gox (72/302, 23.8%). Most HCWs (177/302, 58.6%) attended vaccination training more than once in the past 3 years.

**TABLE 1:** Healthcare workers' characteristics (*n* = 302).

	*n* ^a^	%^a^
Sex		
Women	251	83.1
Men	51	16.9
Age		
Mean (SD)	38.6 (9.1)	
≤30	70	23.2
31–44	165	54.6
≥45	67	22.2
Area		
Urban (Dakar)	129	42.7
Rural (Niakhar HDSS/Fatick)	173	57.3
Position		
Community healthcare worker/Badjenu Gox	72	23.8
Nurse/nursing assistant	115	38.1
Head nurse/major nurse	60	19.9
Midwife/midwife assistant	51	16.9
Other	4	1.3
Tenure		
Mean (SD)	7.5 (7.1)	
<3 years	83	27.5
3–9 years	138	45.7
≥10 years	81	26.8
Professional status		
Civil servant	69	22.9
Permanent contract	45	14.9
Fixed‐term contract	110	36.4
Volunteer	78	25.8
Number of patients per week		
<50	108	35.8
51–100	129	42.7
>100	65	21.5
Estimated workload		
Average‐low	110	36.4
High	147	48.7
Too high	45	14.9
In the past 3 years, has attended a vaccination training seminar		
More than once	177	58.6
Once	61	20.2
No	64	21.2

^a^ Unless otherwise specified.

### Vaccine recommendation practices

Table 2 presents the six items used to construct the vaccine recommendation score. Specifically, 77.8% (140/180) of HCWs systematically (“always”) recommended the tetanus vaccine to mothers, 89.5% (257/287) consistently recommended the measles, rubella, and yellow fever vaccines, and 85.5% (200/234) consistently recommended the hepatitis B vaccine to infants. Regarding HPV vaccination for girls aged 9 to 14, 88.1% (266/302) of HCWs “always” recommended the vaccine when interacting with mothers of unvaccinated daughters. Almost two third of HCWs (183/302, 60.6%) achieved the maximum vaccine recommendation score (Data S2).

**TABLE 2:** Healthcare workers' vaccine recommendation items (*n* = 302).

	*n*	%	Cronbach's alpha coefficient with item deleted^a^
When receiving/visiting mothers who have just given birth and have not received the tetanus vaccine, actively recommend this vaccine			
Do not receive/visit this type of patient	122	40.4	
Don't know	1	0.6	
Never^b^	12	6.7	
Sometimes^b^	10	5.6	
Often^b^	17	9.4	
Always^b^	140	77.8	0.74
When receiving/visiting mothers whose infant has not received the measles, rubella, and yellow fever vaccines, actively recommend vaccinating the infant			
Do not receive/visit this type of patient	15	5.0	
Sometimes^b^	9	3.1	
Often^b^	21	7.3	
Always^b^	257	89.5	0.75
When receiving/visiting mothers whose infant has not received the hepatitis B vaccine, actively recommend vaccinating the infant			
Do not receive/visit this type of patient	68	22.5	
Never^b^	2	0.9	
Sometimes^b^	16	6.8	
Often^b^	16	6.8	
Always^b^	200	85.5	0.75
When receiving/visiting mothers of girls aged 9 to 14 who have not received the human papillomavirus vaccine, actively recommend vaccinating their daughters			
Never	2	0.7	
Sometimes	7	2.3	
Often	27	8.9	
Always	266	88.1	0.73
In 2021–2022, when receiving/visiting adults under 60 who had not received a COVID‐19 vaccine, actively recommend the vaccine			
Don't know	1	0.3	
Never	5	1.7	
Sometimes	12	4.0	
Often	43	14.2	
Always	241	79.8	0.75
In 2021–2022, when receiving/visiting adults over 60 who had not received a COVID‐19 vaccine, actively recommend the vaccine			
Don't know	1	0.3	
Never	5	1.7	
Sometimes	14	4.6	
Often	40	13.3	
Always	242	80.1	0.75

^a^ Global Cronbach's alpha coefficient (6 items): 0.78. For the calculation of Cronbach's alpha, all items were binarized (Always/Not always).

^b^ Percentages are calculated among healthcare workers who reveive/visit this type of patients.

### Vaccine confidence and psychosocial determinants

Overall, HCWs demonstrated low complacency, with 84.4% (255/302) strongly disagreeing that some vaccines are unnecessary (Table 3). A strong sense of collective responsibility was observed, as 95.7% (289/302) strongly agreed that vaccination is essential for community immunity. Trust in authorities was also notably high, with 95.0% (287/302) fully trusting the Ministry of Health to ensure vaccine safety. Nearly three‐quarters (222/302, 73.5%) of HCWs consistently respected patient autonomy by providing vaccine information without exerting undue influence, and 84.4% (255/302) were actively involved in ensuring that patients were vaccinated. The proportion of respondents who perceived the HPV and COVID‐19 vaccines as “very effective” was comparable (69.9% (211/302) and 63.3% (191/302), respectively).

**TABLE 3:** Three‐cluster Pro‐VC‐Be typology of vaccine confidence and other psychosocial determinants among healthcare workers (*n* = 302).

Dimension item	Cluster 1 “Highly confident” (*n* = 173, 57.3%)		Cluster 2 “Moderately hesitant” (*n* = 43, 14.2%)		Cluster 3 “Specific hesitant” (*n* = 86, 28.5%)		All	
	*n*	%	*n*	%	*n*	%	*n*	%
Complacency. Today, Some vaccines recommended by authorities are not useful, because the diseases they prevent are not serious								
Yes, totally agree	7	4.1	3	7.0	1	1.2	11	3.6
Yes, rather agree	0	0.0	0	0.0	1	1.2	1	0.3
Neither agree nor disagree, or don't know	1	0.6	1	2.3	0	0.0	2	0.7
No, rather disagree	2	1.2	29	67.4	2	2.3	33	10.9
No, totally disagree	163	94.2	10	23.3	82	95.4	255	84.4
Perceived collective responsibility. I recommend the vaccines on the vaccination schedule to my patients because it's essential to contribute to protection of the population (community immunity)								
Yes, totally agree	173	100.0	37	86.1	79	91.9	289	95.7
Yes, rather agree	0	0.0	4	9.3	6	7.0	10	3.3
Neither agree nor disagree, or don't know	0	0.0	1	2.3	0	0.0	1	0.3
No, totally disagree	0	0.0	1	2.3	1	1.2	2	0.7
Trust in authorities. I trust the ministry of health to ensure that vaccines are safe								
Yes, totally trust	173	100.0	39	90.7	75	87.2	287	95.0
Yes, rather trust	0	0.0	3	7.0	10	11.6	13	4.3
Neither trust nor distrust, or don't know	0	0.0	1	2.3	0	0.0	1	0.3
No, rather distrust	0	0.0	0	0.0	1	1.2	1	0.3
Openness to patients. I inform my patients about the benefits and risks of vaccines but I let them make their decision without trying to influence them								
Don't know	0	0.0	0	0.0	1	1.2	1	0.3
Never	14	8.1	14	32.6	3	3.5	31	10.3
Sometimes	15	8.7	3	7.0	5	5.8	23	7.6
Often	11	6.4	13	30.2	1	1.2	25	8.3
Always	133	76.9	13	30.2	76	88.4	222	73.5
Commitment to vaccination. I am actively involved in ensuring that my patients are vaccinated								
Never	1	0.6	0	0.0	0	0.0	1	0.3
Sometimes	2	1.2	4	9.3	1	1.2	7	2.3
Often	3	1.7	31	72.1	5	5.8	39	12.9
Always	167	96.5	8	18.6	80	93.0	255	84.4
Self‐efficacy. I feel sufficiently trained on how to approach the question of vaccines with hesitant patients								
Never	0	0.0	1	2.3	0	0.0	1	0.3
Sometimes	3	1.7	9	20.9	6	7.0	18	6.0
Often	10	5.8	21	48.8	8	9.3	39	12.9
Always	160	92.5	12	27.9	72	83.7	244	80.8
Reluctant trust. I recommend the vaccines in the official schedule even though I feel that the objectives of the vaccination policy are not clear enough								
Don't know	0	0.0	0	0.0	2	2.3	2	0.7
Never	103	59.5	30	69.8	70	81.4	203	67.2
Sometimes	20	11.6	5	11.6	6	7.0	31	10.3
Often	5	2.9	5	11.6	2	2.3	12	4.0
Always	45	26.0	3	7.0	6	7.0	54	17.9
Perceived benefit. The human papillomavirus virus (HPV) vaccine effectively protects against this disease								
Don't know	14	8.1	0	0.0	15	17.4	29	9.6
Yes, this vaccine is very effective	159	91.9	33	76.7	19	22.1	211	69.9
Yes, this vaccine is rather effective	0	0.0	10	23.3	52	60.5	62	20.5
Perceived benefit. The COVID‐19 vaccines available in Senegal effectively protect against this disease								
Don't know	1	0.6	0	0.0	0	0.0	1	0.3
Yes, these vaccines are very effective	161	93.1	24	55.8	6	7.0	191	63.3
Yes, these vaccines are rather effective	10	5.8	17	39.5	74	86.1	101	33.4
No, these vaccines are rather ineffective	1	0.6	2	4.7	6	7.0	9	3.0
Perceived constraints. Abstain from vaccinating patients due to material issues								
Never	104	60.1	36	83.7	69	80.2	209	69.2
Sometimes	64	37.0	6	14.0	15	17.4	85	28.2
Often	5	2.9	1	2.3	2	2.3	8	2.7

The HCA yielded three distinct clusters of HCWs (Table 3). Cluster 1 (57.3%, 173/302) represents HCWs with the highest confidence and commitment toward vaccination. Specifically, 94.2% (163/173) of this group strongly rejected complacency regarding the importance of vaccines, and all (100%) trusted health authorities and exhibited a strong agreement with collective responsibility. They felt adequately trained to handle vaccine‐hesitant patients. This cluster also perceived the HPV and COVID‐19 vaccines as very effective (91.9% (159/1673) and 93.1% (161/173), respectively). We designated this cluster as the ‘Highly Confident’. Cluster 2 (14.2%, 43/302) displayed moderate levels of vaccine confidence, with some hesitancy. HCWs in this cluster showed higher levels of uncertainty, as reflected by only 23.3% (10/43) who “totally disagree” that some vaccines are unnecessary. Levels of openness to patients, commitment to vaccination, and self‐efficacy were also significantly lower in this group (30.2% (13/43), 18.6% (8/43), and 27.9% (12/43), respectively, with an “always” response). Compared to the first cluster, this group held mixed views regarding the effectiveness of the HPV and COVID‐19 vaccines, with 76.7% (33/43) and 55.8% (24/43), respectively, not considering these vaccines to be “very effective”. We designated this second cluster as the “Moderately hesitant”. Cluster 3 (28.5%, 86/302) presented the most varied levels of vaccine confidence and engagement. Most members (95.4%, 82/86) “totally disagreed” with vaccine complacency. Openness to patients and reluctant trust were more prevalent in this cluster (88.4% (76/86) and 81.4% (70/86), respectively). However, the perceived effectiveness of the HPV and COVID‐19 vaccines was substantially lower compared to clusters 1 and 2, with only 22.1% (19/86) and 7.0% (6/86), respectively, rating these vaccines as “very effective.” This is why we designated this last cluster as the “Specific hesitant”. A detailed description of the three clusters is available in Data S3.

### Factors associated with systematic vaccine recommendations

The bivariate analysis identified several factors significantly associated with achieving the highest vaccine recommendation score (Table 4). Women, urban HCWs, those with over 3 years of tenure, and nurses/nurse assistants were more likely to achieve the highest score. A significant non‐linear association was also found with workload. Regarding the Pro‐VC‐Be typology, the proportion of HCWs achieving the highest vaccine recommendation score was highest in the “Highly Confident” cluster (69.4% (120/173) vs. 48.8% (63/129), *p* < 0.001) and the lowest in the “Moderately Hesitant” cluster (23.3% (10/43) vs. 66.8% (173/259), *p* < 0.001). After the stepwise selection procedure in the log‐binomial multivariable model (Table 4), HCWs with more than 3 years of tenure (risk ratio [RR]: 1.30, 95% confidence interval [CI]: 1.03–1.63, *p* = 0.025) and those working in urban areas (RR: 1.28, 95% CI: 1.08–1.52, *p* = 0.005) had a higher likelihood of achieving the highest vaccine recommendation score, all else being equal. Conversely, HCWs in the “Moderately Hesitant” cluster had a significantly lower probability of achieving the highest score compared to those in the “Highly Confident” cluster (RR: 0.39, 95% CI: 0.22–0.69, *p* = 0.001). Among the three explanatory variables included, the Pro‐VC‐Be typology made the largest contribution to the model (χ^2^ = 17.93, *p* = 0.001).

**TABLE 4:** Factors associated with the vaccine recommendation score: Multivariable log‐binomial regression (*n* = 302).

	Low vaccine recommendation score (*n* = 119, 39.4%)		High vaccine recommendation score (*n* = 183, 60.6%)		Multivariable log‐binomial stepwise regression	
	*n*	%	*n*	%	Risk ratio (95% CI)	*p*‐value
Sex						
Women	92	36.7	159	63.4		
Men	27	52.9	24	47.1*	NS	
Age						
≤30	33	47.1	37	52.9		
>30	86	37.1	146	62.9		
Area						
Rural (Niakhar HDSS/Fatick)	86	49.7	87	50.3	Ref.	
Urban (Dakar)	33	25.6	96	74.4***	1.28 (1.08–1.52)	0.005
Position						
Other	85	45.5	102	54.6		
Nurse/Nursing Assistant	34	29.6	81	70.4**	NS	
Tenure						
<3 years	43	51.8	40	48.2	Ref.	
≥3 years	76	34.7	143	65.3**	1.30 (1.03–1.63)	0.025
Professional status						
Other	83	37.1	141	63.0		
Volunteer/motivation	36	46.2	42	53.9		
Number of patients per week						
<50	48	44.4	60	55.6		
51–100	44	34.1	85	65.9		
>100	27	41.5	38	58.5		
Estimated workload						
Average‐low	49	44.6	61	55.5		
High	48	32.7	99	67.4*	NS	
Too high	22	48.9	23	51.1		
In the past 3 years, has attended a vaccination training seminar						
No/Yes, once	55	44.0	70	56.0		
Yes, several times	64	36.2	113	63.8		
Pro‐VC‐Be Typology						
Cluster 1 “Highly confident”	53	30.6	120	69.4***	Ref.	
Cluster 2 “Moderately hesitant”	33	76.7	10	23.3***	0.39 (0.22–0.69)	0.001
Cluster 3 “Specific hesitant”	33	38.4	53	61.6	0.90 (0.75–1.08)	0.266

*Note*: *p < 0.05, **p < 0.01 and ***p < 0.001, respectively (Fisher's exact test).

Abbreviation: NS: Non statistically significant after stepwise selection.

## DISCUSSION

This study of HCWs in Senegal showed a high overall vaccine confidence and proactive behaviours in recommending vaccines, demonstrating a strong commitment to vaccinating their patients across a range of diseases. HCWs with more than 3 years of experience and those working in urban areas were significantly more likely to consistently recommend vaccination. Conversely, the Pro‐VC‐Be typology was the strongest factor associated with the recommendation score: moderately hesitant HCWs, who displayed greater complacency toward vaccines, along with lower levels of openness to patients, commitment to vaccination, and self‐efficacy, were less likely to consistenly recommend vaccination.

The high level of proactive vaccine promotion observed among HCWs in this study is noteworthy, with nearly two‐thirds achieving the maximum vaccine recommendation score. Moreover, in comparison to other studies using the Pro‐VC‐Be questionnaire [20, 23], HCWs in this study demonstrated greater confidence in and commitment to vaccination. These findings contrast with the well‐documented vaccine hesitancy among HCWs in the region, particularly concerning the COVID‐19 vaccine [32, 33]. In our study, 80% of HCWs reported ‘always’ recommending the COVID‐19 vaccine to their patients, and 97% described it as ‘effective’ or ‘very effective’. By comparison, a systematic review and meta‐analysis in SSA [34] estimated the pooled prevalence of COVID‐19 vaccine hesitancy among HCWs at 46% (95% CI: 0.38–0.54). Similarly, for the HPV vaccine, introduced into Senegal's EPI in 2018, a community‐level cross‐sectional survey conducted in Senegal in 2020 reported a 72% recommendation rate among HCWs lower than the 88% observed in our study [12]. However, direct comparisons with existing literature are challenging due to differences in study settings, the methodologies used to estimate vaccine hesitancy among HCWs, and the vaccines examined. The divergence in findings with other SSA regions may also be attributed to the distinctive context of Senegal's immunisation program. Regular training sessions and the collaborative involvement of civil society organisations, such as Badjenu Gox, likely contribute to enhancing HCWs' sense of responsibility and fostering their proactive engagement in promoting vaccination [9, 26]. Badjenu Gox are typically experienced women who are well‐respected and influential within their communities. Recruited and trained by the health system, they serve as mediators between the health system and the population [26]. For example, during HPV vaccination campaigns, Badjenu Gox play a critical role in informing and sensitising parents, as well as obtaining their consent before vaccination teams visit schools.

Our findings also reveal notable disparities between HCWs in urban and rural areas, with urban HCWs being significantly more likely to recommend vaccines. A plausible explanation is that HCWs in urban settings may have better access to training opportunities, greater availability of resources, and more frequent exposure to updated vaccination guidelines. These factors are often associated with an increased likelihood of promoting vaccination [35, 36, 37]. However, our study found no significant association between participation in a training programme within the past 3 years and vaccine recommendation. This unexpected result could suggest that the training variable in our study may not adequately reflect HCWs' actual knowledge or skills. Instead, the rural/urban variable may serve as a more robust proxy for differences in access to information and the quality of training programmes. Furthermore, higher patient volumes in urban areas could encourage HCWs to adopt more proactive vaccine promotion practices. In contrast, rural HCWs frequently encounter greater logistical and infrastructural challenges than their urban counterparts, which can hinder consistent participation in vaccination initiatives [38, 39]. Another contributing factor could be that rural HCWs encounter more vaccine‐hesitant patients, influenced by deeply rooted cultural beliefs, misinformation, or lower health literacy levels [40, 41]. Such interactions may discourage HCWs from consistently recommending vaccines, particularly if they lack adequate tools and institutional support to address vaccine hesitancy effectively. In the context of COVID‐19, the higher vaccine recommendation rates observed among urban HCWs may seem counterintuitive. Some studies have reported that urban areas in SSA, despite having better access to healthcare services, exhibit higher levels of COVID‐19 related vaccine hesitancy [42, 43]. Factors such as the rapid dissemination of misinformation through social media and lower trust in government‐led health initiatives are thought to contribute to this higher vaccine hesitancy in urban areas. However, these findings largely stem from studies on the general population, not HCWs, and may not be directly applicable to other vaccines.

Another key finding of this study is the strong association between the Pro‐VC‐Be typology and the likelihood of HCWs recommending vaccination. Specifically, HCWs within the “moderately hesitant” cluster—characterised by elevated vaccine complacency, along with lower levels of openness to patients, commitment to vaccination, and self‐efficacy—were less likely to consistently recommend vaccination. These findings align with previous research using the Pro‐VC‐Be questionnaire [20, 23, 44], which identified self‐efficacy and commitment as the dimensions most strongly associated with HCWs' vaccine recommendations to their patients. These dimensions, which have been both theorised and empirically validated as key determinants of preventive behaviours promoted by HCWs [45], are essential psychosocial resources that enable HCWs to advocate for vaccination among their patients. After accounting for area of residence and seniority, HCWs in the “Specific hesitant” cluster did not exhibit a significantly lower vaccine recommendation score than those in the “confidents” cluster. This finding is somewhat unexpected, as vaccine recommendations are often strongly associated with the perceived effectiveness of the vaccine [46]. However, similar to the general population, vaccine hesitancy among HCWs is highly context‐specific, varying significantly across different diseases and vaccines [47, 48]. Consequently, HPV or COVID‐19 related vaccine hesitancy may not necessarily translate into lower recommendations for other long‐established routine vaccines [49, 50].

## LIMITATIONS

As the Niakhar area has been a site of extensive research over several years, particularly in the fields of infectious disease epidemiology and infectiology, HCWs practicing in this region may not be representative of other rural populations in Senegal. However, despite the intense health surveillance in the Niakhar area, childhood vaccination coverage is not significantly higher than in other rural areas of Senegal [51]. Similarly, although the random selection of health posts in the urban area helps reduce selection bias, HCWs working in these health posts may differ from those in other urban settings, which may also limit the generalisability of our findings. Within each health post, HCWs were not randomly selected but were limited to those present during interviewer visits. While this may affect generalisability, the very high response rate (99.0%) and the likelihood that absent HCWs did not differ systematically from those present suggest that the risk of selection bias is limited. Social desirability bias cannot be ruled out, as numerous studies have documented significant vaccine hesitancy among HCWs in SSA, particularly regarding the COVID‐19 vaccine [34]. Another limitation is that the 5‐point Likert scale used only assessed the frequency of positive vaccine recommendations and did not allow for the reporting of negative or discouraging advice. Thus, we may have missed HCWs who actively discouraged vaccination. While some HCWs are known to spread vaccine misinformation [52, 53], the very low proportion of HCWs in our sample reporting never recommending vaccination suggests that the risk of missing such cases is minimal. Finally, the cross‐sectional design limits the ability to infer causality between HCWs characteristics and vaccine recommendation behaviours.

## CONCLUSION

This study highlights the high levels of vaccine confidence and proactive recommendation behaviours among HCWs in Senegal. However, disparities between rural and urban HCWs, as well as the differences found among HCWs regarding some psychosocial factors such as complacency and self‐efficacy, underscore the need for targeted interventions. In particular, in rural settings, where training opportunities and resources are often limited, interventions should focus on improving access to continuous professional development and providing practical tools for addressing vaccine hesitancy. Future research should further explore contextual factors influencing HCWs' practices to guide tailored and effective strategies. This study also enabled the adaptation of Pro‐VC‐Be to an African context and demonstrated its relevance for future research in Senegal and accross the continent. By addressing these challenges, policymakers and health authorities can strengthen HCWs' capacity to promote vaccines and sustain high immunisation coverage across diverse healthcare settings.

## AUTHOR CONTRIBUTIONS

Conceptualization: Sébastien Cortaredona, Pierre Verger and Patrick Peretti‐Watel; Formal analysis: Sébastien Cortaredona; Investigation (data collection): Gwenaelle Maradan, El‐Hadj Ba and Cheikh Sokhna; Writing—Original Draft: Sébastien Cortaredona, Pierre Verger and Patrick Peretti‐Watel. Writing—Reviewing & Editing: All authors. Supervision, Project administration and Funding acquisition: Patrick Peretti‐Watel and Cheikh Sokhna.

## FUNDING INFORMATION

This study is supported by the French National Research Agency (ANR‐20‐CE36‐0005‐01).

## CONFLICT OF INTEREST STATEMENT

The authors have no relevant financial or non‐financial relationships to disclose.

## Supporting information


**DATA S1.** Supporting Information.

**DATA S2.** Supporting Information.

**DATA S3.** Supporting Information.

## Data Availability

The datasets generated during and/or analysed during the current study are available from the corresponding author on reasonable request.
