# Supplementary material for: Understanding vaccine recommendation behaviours among healthcare workers in Senegal: A cross‐sectional analysis
Source: Trop Med Int Health. 2025 Jun 29;30(8):853–64. doi: 10.1111/tmi.70002 (PMC12318442; doi:10.1111/tmi.70002)
Supplement: Supplementary file 1 — DATA S1. Supporting Information. [file TMI-30-853-s003.docx]

Understanding Vaccine Recommendation Behaviors among Healthcare Workers in Senegal: A Cross-Sectional Analysis

Sébastien Cortaredona^1,2,3^, Pierre Verger^4,5^, Jean Constance^5^, Aldiouma Diallo^6,7^, El-Hadj Ba^8^, Gwenaelle Maradan^5^, Cheikh Sokhna^1,2,3^, Patrick Peretti-Watel^4,5^

1. Aix-Marseille Univ, IRD, SSA, MINES, Marseille, France.

2. Aix Marseille Univ, SSA, RITMES, Marseille, France

3. IHU-Méditerranée Infection, Marseille, France

4. Unité des Virus Émergents (UVE: Aix-Marseille Univ, Università di Corsica, IRD 190, Inserm 1207, IRBA), Marseille, France.

5. Observatoire régional de la santé PACA (ORS Paca), Aix-Marseille Université, Marseille, France.

6. Comité national d’éthique pour la recherche en santé (CNERS), Dakar, Senegal

7. Conseil consultatif sur les vaccins au Sénégal (CCVS), Dakar, Senegal

8. IRD, MINES, Campus International IRD-UCAD, Dakar, Senegal.

*Corresponding author*

Sébastien Cortaredona

IHU-Méditerranée Infection, 19-21 Bd Jean Moulin, 13005 Marseille

https://orcid.org/0000-0003-3523-7158

[Sebastien.cortaredona@ird.fr](mailto:Sebastien.cortaredona@ird.fr)

**Supplementary file** **1. Final short-form Pro-VC-Be tool to measure vaccine confidence and other psychosocial determinants in healthcare workers^†^.**

| **Dimension** | **Item** |
| --- | --- |
| 1. Perceived risks of vaccines | Some vaccines can cause autoimmune diseases |
| 2. Complacency | Today, some vaccines recommended by authorities are not useful, because the diseases they prevent are not serious |
| 3. Perceived benefit/risk balance | The benefits of vaccines are much greater than their potential risk |
| 4. Perceived collective responsibility | I recommend the vaccines on the vaccination schedule to my patients because it's essential to contribute to protection of the population (community immunity) |
| 5. Trust in authorities | I trust the ministry of health to ensure that vaccines are safe |
| 6. Perceived constraints | The cost of some vaccines is a problem for some patients and can keep me from prescribing them |
| 7. Openness to patients | I inform my patients about the benefits and risks of vaccines but I let them make their decision without trying to influence them |
| 8. Commitment to vaccination | I am actively involved in ensuring that my patients are vaccinated |
| 9. Self-efficacy | I feel sufficiently trained on how to approach the question of vaccines with hesitant patients |
| 10. Reluctant trust | I recommend the vaccines in the official schedule even though I feel that the objectives of the vaccination policy are not clear enough |
| †: The following response scale applies to all items: strongly disagree, somewhat disagree, undecided, somewhat agree, strongly agree | |
