# Supplementary material for: Understanding vaccine recommendation behaviours among healthcare workers in Senegal: A cross‐sectional analysis
Source: Trop Med Int Health. 2025 Jun 29;30(8):853–64. doi: 10.1111/tmi.70002 (PMC12318442; doi:10.1111/tmi.70002)

Understanding Vaccine Recommendation Behaviors among Healthcare Workers in Senegal: A Cross-Sectional Analysis

Sébastien Cortaredona^1,2,3^, Pierre Verger^4,5^, Jean Constance^5^, Aldiouma Diallo^6,7^, El-Hadj Ba^8^, Gwenaelle Maradan^5^, Cheikh Sokhna^1,2,3^, Patrick Peretti-Watel^4,5^

1. Aix-Marseille Univ, IRD, SSA, MINES, Marseille, France.

2. Aix Marseille Univ, SSA, RITMES, Marseille, France

3. IHU-Méditerranée Infection, Marseille, France

4. Unité des Virus Émergents (UVE: Aix-Marseille Univ, Università di Corsica, IRD 190, Inserm 1207, IRBA), Marseille, France.

5. Observatoire régional de la santé PACA (ORS Paca), Aix-Marseille Université, Marseille, France.

6. Comité national d’éthique pour la recherche en santé (CNERS), Dakar, Senegal

7. Conseil consultatif sur les vaccins au Sénégal (CCVS), Dakar, Senegal

8. IRD, MINES, Campus International IRD-UCAD, Dakar, Senegal.

*Corresponding author*

Sébastien Cortaredona

IHU-Méditerranée Infection, 19-21 Bd Jean Moulin, 13005 Marseille

https://orcid.org/0000-0003-3523-7158

[Sebastien.cortaredona@ird.fr](mailto:Sebastien.cortaredona@ird.fr)

**Supplementary file 2. Healthcare workers' vaccine recommendation score based on multiple correspondence analysis first component (n=302).**

| **Item** | **Coordinate on first component** | |
| --- | --- | --- |
|  | **Always (1)** | **Never/**  **Sometimes/**  **Often (0)** |
| When receiving/visiting mothers who have just given birth and have not received the tetanus vaccine, actively recommend this vaccine | 0.34 | -1.61 |
| When receiving/visiting mothers of girls aged 9 to 14 who have not received the human papillomavirus vaccine, actively recommend vaccinating their daughters | 0.29 | -2.16 |
| When receiving/visiting mothers whose infant has not received the measles, rubella, and yellow fever vaccines, actively recommend vaccinating the infant | 0.26 | -1.98 |
| When receiving/visiting mothers whose infant has not received the hepatitis B vaccine, actively recommend vaccinating the infant | 0.26 | -1.84 |
| In 2021-2022, when receiving/visiting adults under 60 who had not received a COVID-19 vaccine, actively recommend the vaccine | 0.31 | -1.24 |
| In 2021-2022, when receiving/visiting adults over 60 who had not received a COVID-19 vaccine, actively recommend the vaccine | 0.3 | -1.22 |
|  |  |  |
|  |  |  |
| \|  \| \| --- \| |  |  |
|  |  |  |
|  |  |  |
|  |  |  |
|  |  |  |
|  |  |  |
|  |  |  |
|  |  |  |
|  |  |  |
|  |  |  |

**Distribution of the vaccine recommendation score**


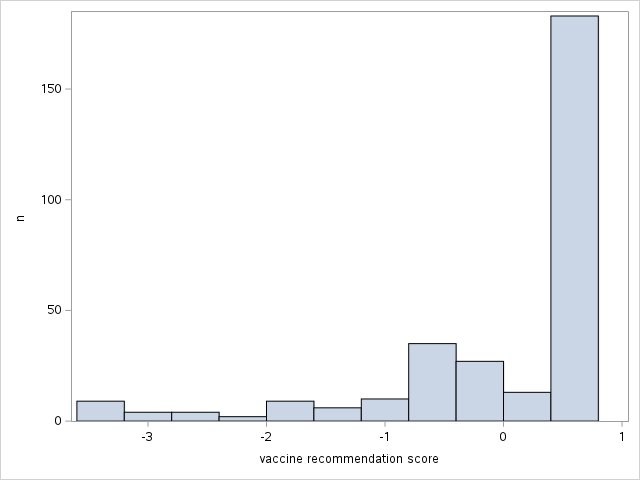

Supplement: Supplementary file 2 — DATA S2. Supporting Information. [file TMI-30-853-s001.docx]
