# Supplementary material for: Understanding vaccine recommendation behaviours among healthcare workers in Senegal: A cross‐sectional analysis
Source: Trop Med Int Health. 2025 Jun 29;30(8):853–64. doi: 10.1111/tmi.70002 (PMC12318442; doi:10.1111/tmi.70002)
Supplement: Supplementary file 3 — DATA S3. Supporting Information. [file TMI-30-853-s002.docx]

Understanding Vaccine Recommendation Behaviors among Healthcare Workers in Senegal: A Cross-Sectional Analysis

Sébastien Cortaredona^1,2,3^, Pierre Verger^4,5^, Jean Constance^5^, Aldiouma Diallo^6,7^, El-Hadj Ba^8^, Gwenaelle Maradan^5^, Cheikh Sokhna^1,2,3^, Patrick Peretti-Watel^4,5^

1. Aix-Marseille Univ, IRD, SSA, MINES, Marseille, France.

2. Aix Marseille Univ, SSA, RITMES, Marseille, France

3. IHU-Méditerranée Infection, Marseille, France

4. Unité des Virus Émergents (UVE: Aix-Marseille Univ, Università di Corsica, IRD 190, Inserm 1207, IRBA), Marseille, France.

5. Observatoire régional de la santé PACA (ORS Paca), Aix-Marseille Université, Marseille, France.

6. Comité national d’éthique pour la recherche en santé (CNERS), Dakar, Senegal

7. Conseil consultatif sur les vaccins au Sénégal (CCVS), Dakar, Senegal

8. IRD, MINES, Campus International IRD-UCAD, Dakar, Senegal.

*Corresponding author*

Sébastien Cortaredona

IHU-Méditerranée Infection, 19-21 Bd Jean Moulin, 13005 Marseille

https://orcid.org/0000-0003-3523-7158

[Sebastien.cortaredona@ird.fr](mailto:Sebastien.cortaredona@ird.fr)

**Supplementary file 3. Characteristics of the three-Cluster Pro-VC-Be typology (n=302).**

|  | **Cluster 1 "Highly confident"  (n=173,  57.3%)** | | **Cluster 2 "Moderately hesitant"  (n=43,  14.2%)** | | **Cluster 3 "Specific hesitant"  (n=86,  28.5%)** | |
| --- | --- | --- | --- | --- | --- | --- |
|  | **n** | **%** | **n** | **%** | **n** | **%** |
| **Sex** |  |  |  |  |  |  |
| Women | 146 | 84.4 | 30 | 69.8 | 75 | 87.2 |
| Men | 27 | 15.6 | 13 | 30.2 | 11 | 12.8 |
| **Age** |  |  |  |  |  |  |
| ≤30 | 34 | 19.7 | 15 | 34.9 | 21 | 24.4 |
| 31-44 | 98 | 56.7 | 21 | 48.8 | 46 | 53.5 |
| ≥45 | 41 | 23.7 | 7 | 16.3 | 19 | 22.1 |
| **Area** |  |  |  |  |  |  |
| Urban (Dakar) | 83 | 48.0 | 0 | 0.0 | 46 | 53.5 |
| Rural (Niakhar HDSS/Fatick) | 90 | 52.0 | 43 | 100.0 | 40 | 46.5 |
| **Position** |  |  |  |  |  |  |
| Community healthcare worker/Badjenu Gox | 44 | 25.4 | 15 | 34.9 | 13 | 15.1 |
| Nurse/Nursing Assistant | 62 | 35.8 | 9 | 20.9 | 44 | 51.2 |
| ICP/Major Nurse | 32 | 18.5 | 8 | 18.6 | 20 | 23.3 |
| Midwife/Midwife Assistant | 31 | 17.9 | 11 | 25.6 | 9 | 10.5 |
| Other | 4 | 2.3 | 0 | 0.0 | 0 | 0.0 |
| **Tenure** |  |  |  |  |  |  |
| <3 years | 46 | 26.6 | 16 | 37.2 | 23 | 26.7 |
| 3-9 years | 76 | 43.9 | 17 | 39.5 | 45 | 52.3 |
| ≥10 years | 51 | 29.5 | 10 | 23.3 | 18 | 20.9 |
| **Professional Status** |  |  |  |  |  |  |
| Civil servant | 38 | 22.0 | 8 | 18.6 | 23 | 26.7 |
| Permanent contract | 28 | 16.2 | 6 | 14.0 | 11 | 12.8 |
| Fixed-term contract | 77 | 44.5 | 6 | 14.0 | 27 | 31.4 |
| Volunteer / motivation | 30 | 17.3 | 23 | 53.5 | 25 | 29.1 |
| **Number of patients per week** |  |  |  |  |  |  |
| <50 | 58 | 33.5 | 19 | 44.2 | 31 | 36.1 |
| 51-100 | 76 | 43.9 | 17 | 39.5 | 36 | 41.9 |
| >100 | 39 | 22.5 | 7 | 16.3 | 19 | 22.1 |
| **Estimated workload** |  |  |  |  |  |  |
| Average-low | 64 | 37.0 | 20 | 46.5 | 26 | 30.2 |
| High | 79 | 45.7 | 21 | 48.8 | 47 | 54.7 |
| Too high | 30 | 17.3 | 2 | 4.7 | 13 | 15.1 |
| **In the past three years, has attended a vaccination training seminar** |  |  |  |  |  |  |
| Yes, several times | 111 | 64.2 | 18 | 41.9 | 48 | 55.8 |
| Yes, once | 33 | 19.1 | 10 | 23.3 | 18 | 20.9 |
| No | 29 | 16.8 | 15 | 34.9 | 20 | 23.3 |
